# Human Infection with Avian Influenza A(H10N3) Virus, China, 2024

**DOI:** 10.3201/eid3111.250847

**Published:** 2025-11

**Authors:** Jing Wang, Fuyin Bi, Xiaojuan Luo, Hang Huang, Changwei Liang, Ying Zhao, Weitao He, Ning Kang, Jing Wang, Yu Ju, Guanghua Lan

**Affiliations:** Guangxi Center for Disease Prevention and Control, Nanning, China (J. Wang, F. Bi, X. Luo, H. Huang, W. He, N. Kang, J. Wang, Y. Ju, G. Lan); Guangxi Key Laboratory of Major Infectious Disease Prevention and Control and Biosafety Emergency Response, Guangxi Center for Disease Control and Prevention, Nanning (J. Wang, Y. Ju, G. Lan); Nanning Center for Disease Prevention and Control, Nanning (C. Liang); The First Affiliated Hospital of Guangxi University of Chinese Medicine, Nanning (Y. Zhao)

**Keywords:** Influenza, viruses, zoonoses, respiratory infections, H10N3, China

## Abstract

We describe the clinical symptoms and epidemiologic characteristics of a patient infected with avian influenza A(H10N3) virus in Guangxi Province, China, in December 2024. Whole-genome sequencing showed that the virus was highly homologous to a virus from Yunnan Province. H10 subtype viruses should be monitored for potential zoonotic or reassortant events.

Since avian influenza virus (AIV) subtype H10 was isolated in 1949, >2,000 H10 subtype AIVs have been isolated from wild waterfowl, poultry, and mammals worldwide (*1*). Cross-species spillovers make AIV prevention and control a major One Health challenge (*2*). According to the World Health Organization weekly update on AIV surveillance published December 20, 2024, only 3 human cases of AIV A(H10N3) virus infection had been reported worldwide, all from China (*3*). We report another human case of H10N3 virus infection in Nanning, Guangxi Zhuang Autonomous Region, China.

On December 12, 2024, a 23-year-old woman began experiencing fever (maximum axillary temperature 40°C) and cough. After failed symptomatic management at a local clinic on December 16, she was referred to the hospital for outpatient evaluations on December 17 and 18. Her condition deteriorated, and she was admitted to the hospital on December 19 with severe community-acquired pneumonia complicated by type I respiratory failure. Moreover, clinical blood and biochemical tests showed elevated C-reactive protein (75.8 mg/L; reference range 0.5–10 mg/L) (Appendix Table). 

Chest computed tomography imaging revealed thickened lung markings with patchy areas of high density in both lungs. Because of worsening respiratory failure, the patient was transferred to the respiratory intensive care unit on December 22 for VV-ECMO (venovenous extracorporeal membrane oxygenation) (*4*). 

Reverse transcription PCR of sputum specimens analyzed by the Nanning Centers for Disease Control and Prevention were positive for A(H10N3) AIV on December 23. After >10 days of treatment with VV-ECMO and antiviral drugs, the patient recovered uneventfully and was discharged on February 8 (Figure 1).

**Figure 1:**
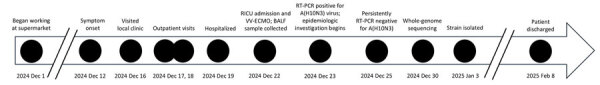
Timeline of disease progression and treatment history in a case of human infection with avian influenza A(H10N3) virus, China, 2024. BALF, bronchoalveolar lavage fluid; RICU, respiratory intensive care unit; RT-PCR, reverse transcription PCR; VV-ECMO, venovenous extracorporeal membrane oxygenation.

The patient had no history of exposure to live poultry before disease onset. She worked in a local supermarket’s meat department that processed and sold fresh pork, beef, and poultry products (chicken and duck), but no live poultry was handled on site. Four close contacts and 12 colleagues of the case-patient completed a 10-day health monitoring period and underwent influenza nucleic acid testing, all negative for H10N3 virus.

Comprehensive environmental surveillance was conducted across critical exposure sites. Swab samples were collected from the patient’s residence (n = 8); occupational environment, poultry supply chain facilities, and network nodes along the poultry supply chain (n = 61); farmers markets adjacent to the patient’s residence (n = 40); and epidemiologically linked locations visited 10 days before symptom onset (n = 23). Among the 132 environmental samples collected, 73 (55.3%) were positive for pan–influenza A virus; subtypes H9 (42.5%, 31/73) and H5 (8.2%, 6/73) predominated. H10 subtype was not detected in any of the samples.

We obtained whole-genome virus sequences isolated from bronchoalveolar lavage fluid and passaged on embryonated chicken eggs. We designated the virus A/Guangxi/01591/2024/H10N3 (GX01591) and submitted full-length sequences of the polymerase basic (PB) 2 (2,341 nt length), PB1 (2,341 nt), polymerase acidic (PA) (2,233 nt), hemagglutinin (HA) (1,728 nt), nucleoprotein (1,565 nt), neuraminidase (NA) (1,452 nt), matrix (M) (1,027), and nonstructural (890 nt) genes to GISAID (https://www.gisaid.org; accession nos. EPI4019311–8). The egg passage sequence was 100% identical to the original sequence. 

We used EpiFlu BLAST from the GISAID influenza database for sequence alignment and downloaded reference sequences. We performed amino acid site analysis with BioEdit 7.01 software (https://thalljiscience.github.io) and constructed a phylogenetic tree with MEGA version 7 (https://www.megasoftware.net). Phylogenetic analysis revealed that the internal genes of GX01591 were closely related to those of A/Yunnan/0110/2024(H10N3) and belonged to the Eurasian AIV lineage (Figure 2). Evolutionary analysis revealed that the HA genes were reassorted from avian-origin H10Nx, NA genes were reassorted from H7N3, and the other 6 internal genes were reassorted from H9N2 influenza viruses (Appendix Figures 1–6).

**Figure 2:**
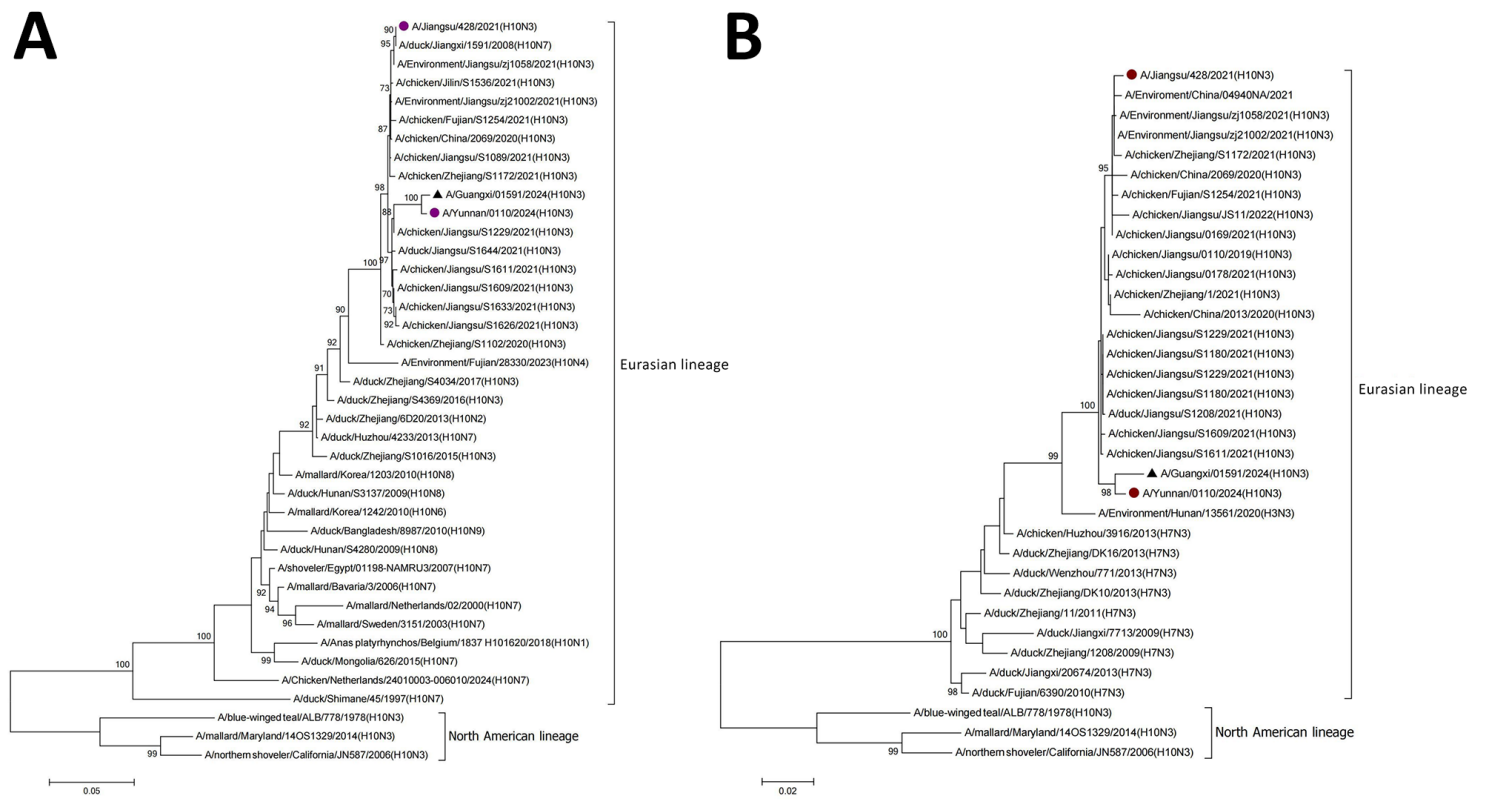
Phylogenetic analysis of avian influenza A(H10N3) virus from human infection, China, 2024. A) Hemagglutinin gene; B) neuraminidase gene. Black triangles indicate A/Guangxi/ 01591/2024(H10N3) virus from patient isolates. Purple circles indicate 2 other virus isolates from humans in China. Scale bar indicates nucleotide substitutions per site.

The HA proteins contained PEIIQGR↓GLLG at the cleavage site, indicating low pathogenicity. The virus had the QSG motif at the receptor binding site (nucleotide positions 226–8), suggesting avian-like receptor specificity (*5*). In addition, residues 95Y, 151W, 183H, 190E, 191K, and 194L of the HA protein indicated that the H10N3 virus could bind to avian-like receptors (*6*). The E119G, H274Y, and R292K molecular markers of NA inhibitors in the NA protein (*7*), and I38T/M/F of the PA inhibitor in the PA protein exhibited no mutations, suggesting susceptibility to the NA inhibitors oseltamivir, zanamivir, and peramivir and to the PA inhibitor baloxavir. We detected an S to N mutation at residue 31 in the M2 protein, indicating resistance to adamantanes. The molecular markers N30D and T215A in the M1 protein and P42S and V149A in the nonstructural 1 protein exhibited mutations, suggesting increased virulence in mice. We also detected the mammalian adaptive mutation D701N in PB2.

As noted in previously reported human infections caused by H10N3 virus (*8*,*9*), our patient initially experienced upper respiratory symptoms before severe pneumonia and respiratory failure developed. Compared with other patients, our patient’s short hospitalization could be attributable to younger age and absence of chronic diseases. GX01591, like other human H10N3 viruses, is an avian-origin reassortant variant (*10*) and has characteristic avian-like receptor specificity, consequently exhibiting low zoonotic transmission risk. However, the AIV H10 virus subtype should be monitored for potential zoonotic or reassortant viruses.

Appendix Additional information on human infection with avian influenza A(H10N3) virus, China, 2024.

## References

[1] Ding S, Zhou J, Xiong J, Du X, Yang W, Huang J, et al. Continued evolution of H10N3 influenza virus with adaptive mutations poses an increased threat to mammals. Virol Sin. 2024;39:546–55. 10.1016/j.virs.2024.06.005 38871182 PMC11401466

[2] Niu Q, Jiang Z, Wang L, Ji X, Baele G, Qin Y, et al. Prevention and control of avian influenza virus: Recent advances in diagnostic technologies and surveillance strategies. Nat Commun. 2025;16:3558. 10.1038/s41467-025-58882-4 40229313 PMC11997231

[3] World Health Organization. Avian influenza weekly update 2024 [cited 2025 Apr 2]. https://iris.who.int/handle/10665/375483.

[4] Teijeiro-Paradis R, Gannon WD, Fan E. Complications associated with venovenous extracorporeal membrane oxygenation—what can go wrong? Crit Care Med. 2022;50:1809–18. 10.1097/CCM.0000000000005673 36094523

[5] Herfst S, Zhang J, Richard M, McBride R, Lexmond P, Bestebroer TM, et al. Hemagglutinin traits determine transmission of avian A/H10N7 influenza virus between mammals. Cell Host Microbe. 2020;28:602–613.e7. 10.1016/j.chom.2020.08.011 33031770 PMC7556738

[6] Zhang M, Zhang X, Xu K, Teng Q, Liu Q, Li X, et al. Characterization of the pathogenesis of H10N3, H10N7, and H10N8 subtype avian influenza viruses circulating in ducks. Sci Rep. 2016;6:34489. 10.1038/srep34489 27678170 PMC5039634

[7] Orozovic G, Orozovic K, Lennerstrand J, Olsen B. Detection of resistance mutations to antivirals oseltamivir and zanamivir in avian influenza A viruses isolated from wild birds. PLoS One. 2011;6:e16028. 10.1371/journal.pone.0016028 21253602 PMC3017088

[8] Jing J, Wang L, Wang G, Dai Z, Ren W, Yi C, et al. A human infection case with avian-origin H10N3 influenza virus. Quant Imaging Med Surg. 2021;11:4508–10. 10.21037/qims-21-592 34604005 PMC8408795

[9] Zhao Z, Luo S, Gao Y, Dai M, Yan J, Yang Y, et al. A case report of human infection with avian influenza H10N3 with a complex respiratory disease history. BMC Infect Dis. 2024;24:918. 10.1186/s12879-024-09830-y 39232670 PMC11373451

[10] Qi X, Qiu H, Hao S, Zhu F, Huang Y, Xu K, et al. Human infection with an avian-origin influenza A (H10N3) virus. N Engl J Med. 2022;386:1087–8. 10.1056/NEJMc2112416 35294820

