# Supplementary material for: Human Infection with Avian Influenza A(H10N3) Virus, China, 2024
Source: Emerg Infect Dis. 2025 Nov;31(11):2174–6. doi: 10.3201/eid3111.250847 (PMC12704535; doi:10.3201/eid3111.250847)
Supplement: Appendix — Additional information on human infection with avian influenza A(H10N3) virus, China, 2024. [file 25-0847-Techapp-s1.pdf]

Article DOI: https://doi.org/10.3201/eid3111.250847

EID cannot ensure accessibility for supplementary materials supplied by authors. Readers who have difficulty accessing supplementary content should contact the authors for assistance.

Human Infection with Avian Influenza A(H10N3) Virus, China, 2024

Appendix

Appendix Table. Clinical blood cell and biochemistry test results from hospital admission to H10N3 positive test*

Tests	19 Dec†	21 Dec	22 Dec‡	23 Dec	24 Dec	Reference range
Leukocytes, cells × 10^9/L	5.52	5.71	5.85	9.81	8.09	3.5–9.5
Lymphocyte, cells × 10^9/L	1.5	1.27	1.15	2.18	1.19	1.1–3.2
Hemoglobin, g/L	140	126	95	101	92	115–150
Neutrophils, %	69.1	74.2	76.8	73.1	77.2	40–75
Lymphocytes, %	27.1	22.2	19.7	22.2	14.7	20–50
Monocyte, %	3.7	3.6	3.3	4.7	8.1	3–10
Eosinophils, %	0	0	0.1	0	0	0.4–8
Basophils, %	0.1	0	0.1	0	0	0.0–1.0
Direct bilirubin, µmol/L	2.2	ND	2.1	5	ND	0–6.8
Indirect bilirubin, µmol/L	3.8	ND	2.2	6.6	ND	2–16.2
Total protein, g/L	66.9	ND	44.8	53.2	ND	65–85
Albumin, g/L	36.3	ND	21.4	34.6	ND	40–55
Globin, g/L	30.6	ND	23.4	18.6	ND	20–40
C-reactive protein, mg/L	75.8	ND	ND	60.7	ND	0.5–10

*ND, not done.

†The patient was admitted to hospital.

‡The patient was transferred to the Respiratory Intensive Care Unit (RICU).

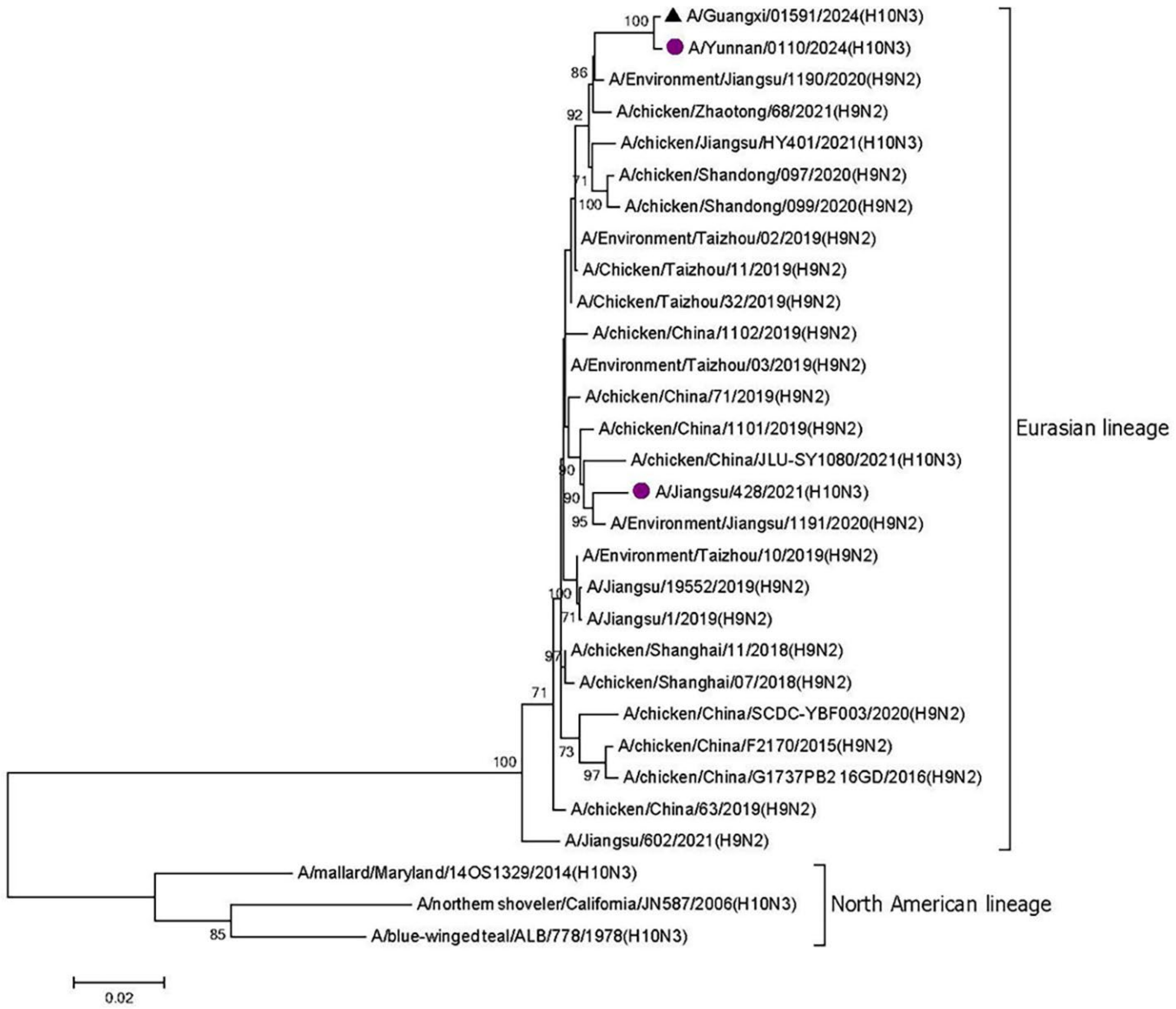

Appendix Figure 1. Phylogenetic tree PB2 gene of the A/Guangxi/01591/2024(H10N3). The phylogenetic trees were constructed with MEGA7.0 software using the maximum likelihood (ML) method. Bootstrap analysis was performed with 1000 replicates. The virus A/Guangxi/01591/2024(H10N3) reported in Guangxi is marked with black triangles. The other 2 viruses isolated from human in People's Republic of China were marked with purple circle. Scale bar indicates nucleotide substitutions per site.

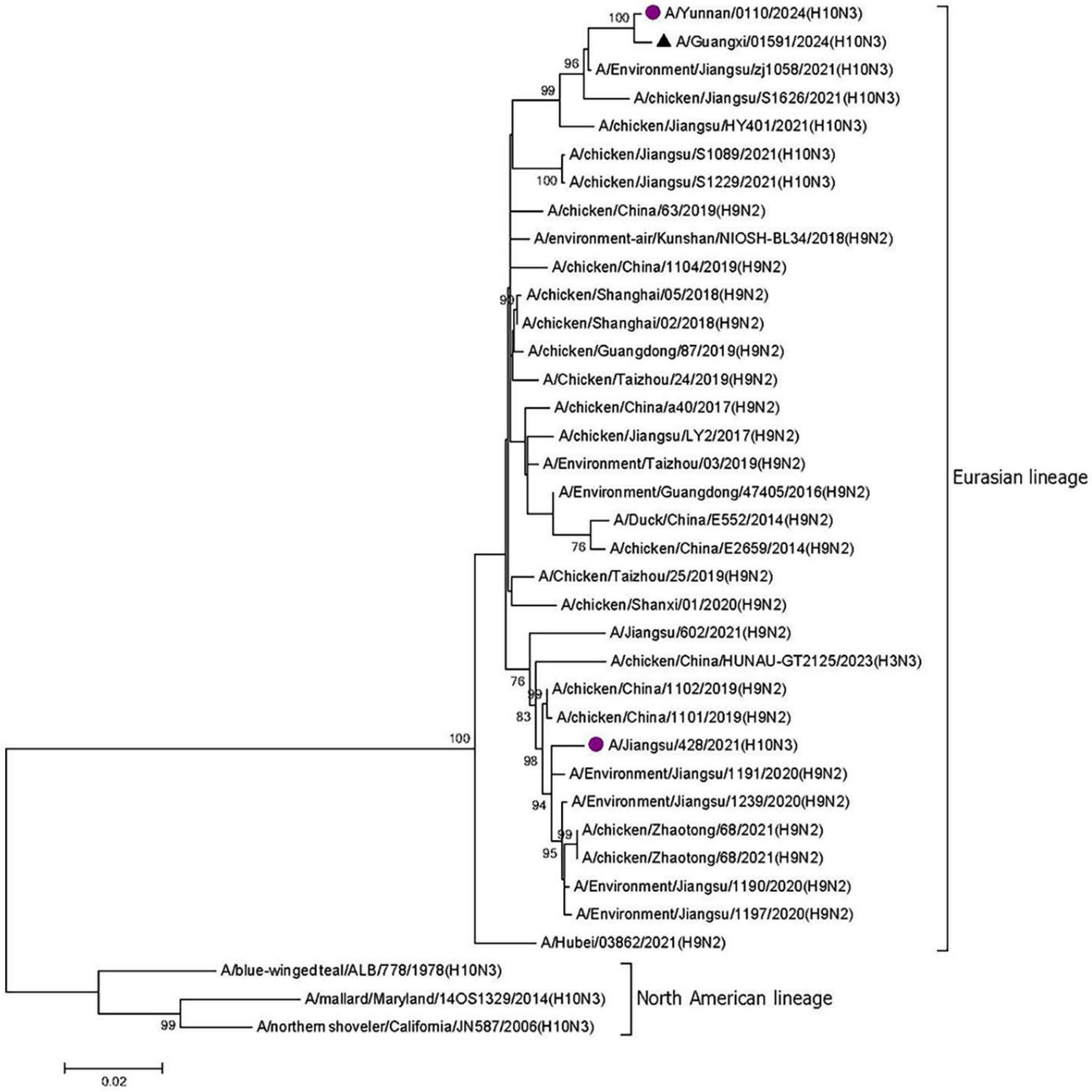

Appendix Figure 2. Phylogenetic tree PB1 gene of the A/Guangxi/01591/2024(H10N3). The phylogenetic trees were constructed with MEGA7.0 software using the maximum likelihood (ML) method. Bootstrap analysis was performed with 1000 replicates. The virus A/Guangxi/01591/2024(H10N3) reported in Guangxi is marked with black triangles. The other 2 viruses isolated from human in People's Republic of China were marked with purple circle. Scale bar indicates nucleotide substitutions per site.

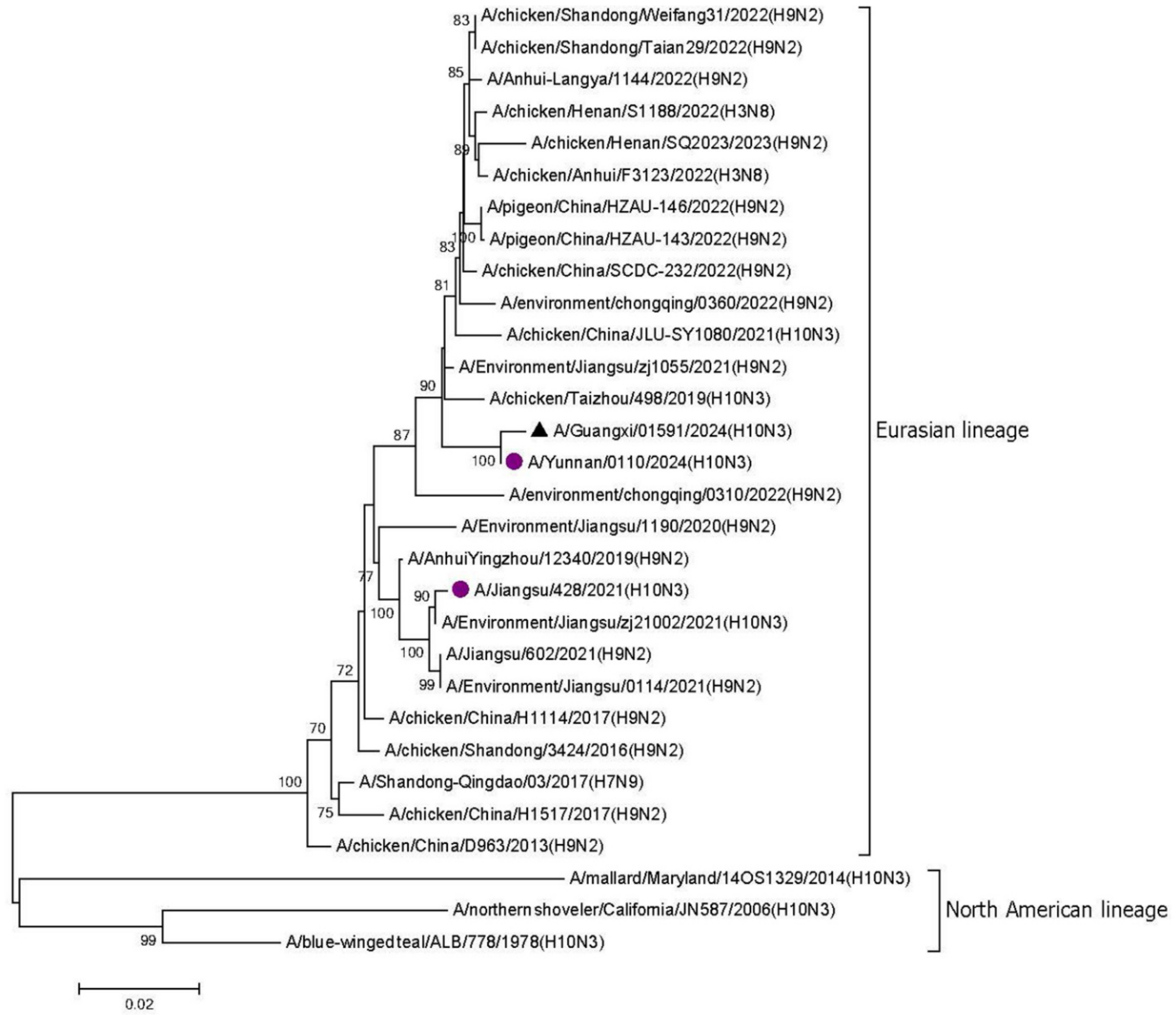

Appendix Figure 3. Phylogenetic tree PA gene of the A/Guangxi/01591/2024(H10N3). The phylogenetic trees were constructed with MEGA7.0 software using the maximum likelihood (ML) method. Bootstrap analysis was performed with 1000 replicates. The virus A/Guangxi/01591/2024(H10N3) reported in Guangxi is marked with black triangles. The other 2 viruses isolated from human in People's Republic of China were marked with purple circle. Scale bar indicates nucleotide substitutions per site.

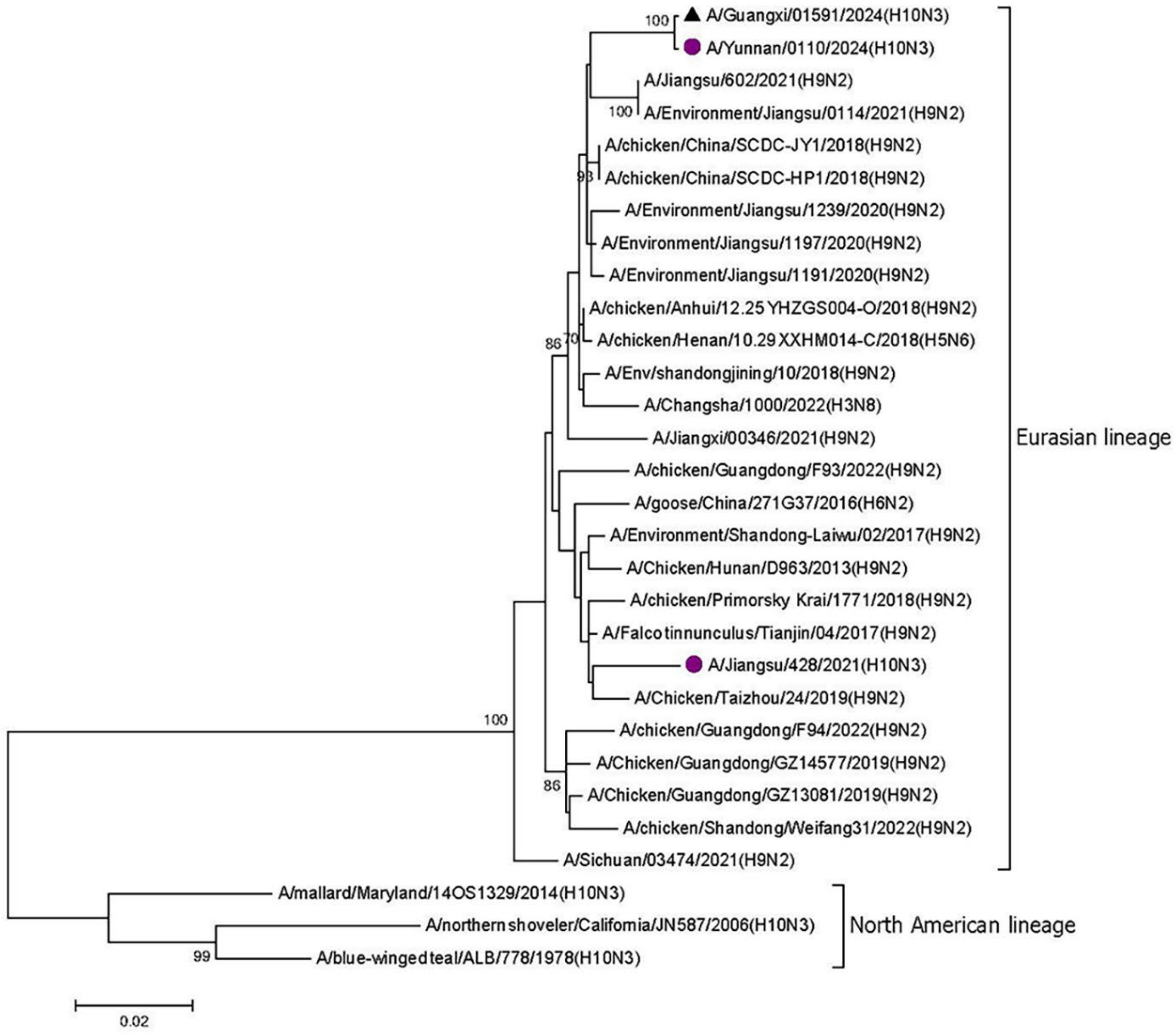

Appendix Figure 4. Phylogenetic tree NP gene of the A/Guangxi/01591/2024(H10N3). The phylogenetic trees were constructed with MEGA7.0 software using the maximum likelihood (ML) method. Bootstrap analysis was performed with 1000 replicates. The virus A/Guangxi/01591/2024(H10N3) reported in Guangxi is marked with black triangles. The other 2 viruses isolated from human in People's Republic of China were marked with purple circle. Scale bar indicates nucleotide substitutions per site.

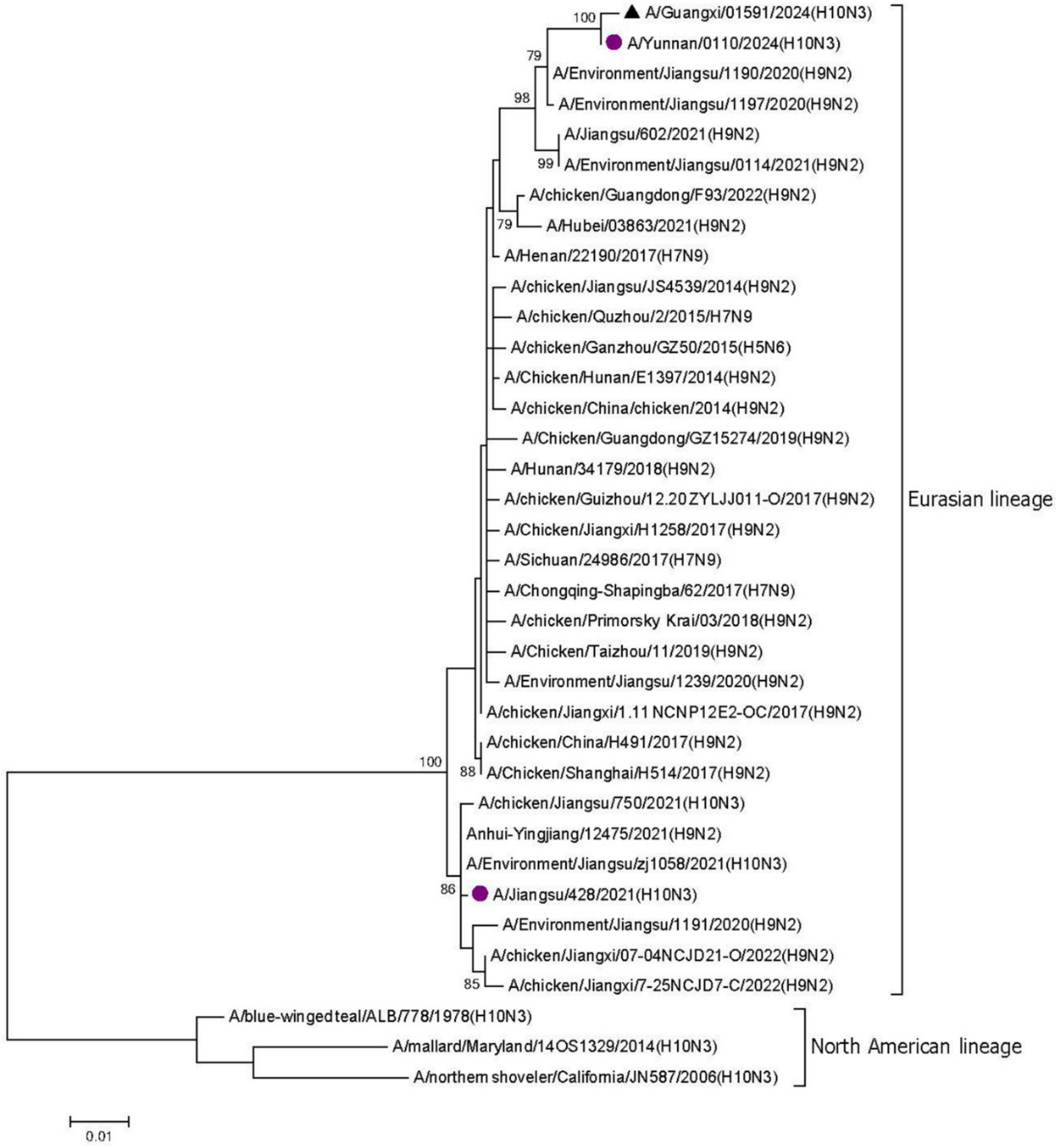

Appendix Figure 5. Phylogenetic tree MP gene of the A/Guangxi/01591/2024(H10N3). The phylogenetic trees were constructed with MEGA7.0 software using the maximum likelihood (ML) method. Bootstrap analysis was performed with 1000 replicates. The virus A/Guangxi/01591/2024(H10N3) reported in Guangxi is marked with black triangles. The other 2 viruses isolated from human in People's Republic of China were marked with purple circle. Scale bar indicates nucleotide substitutions per site.

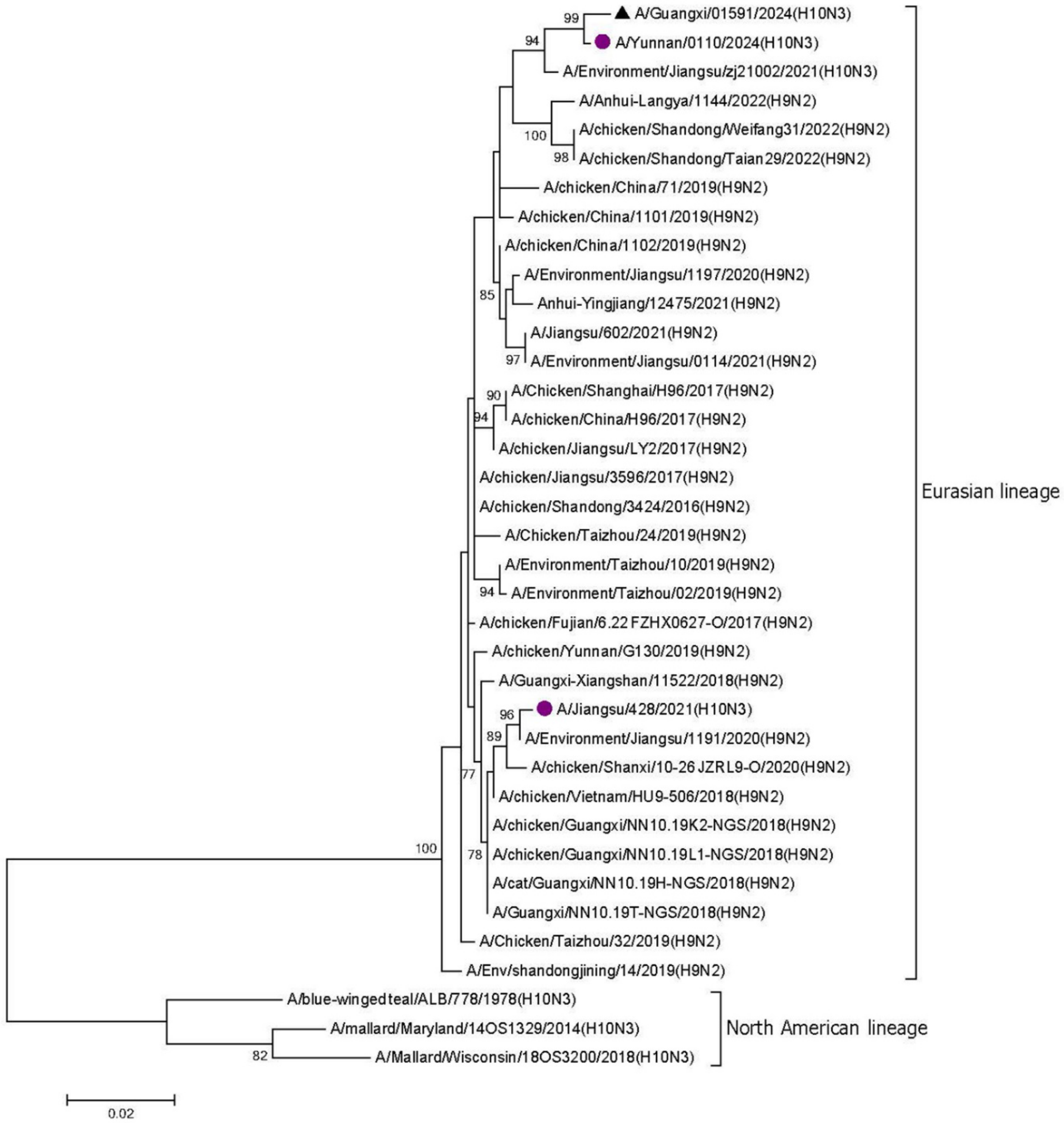

Appendix Figure 6. Phylogenetic tree NS gene of the A/Guangxi/01591/2024(H10N3). The phylogenetic trees were constructed with MEGA7.0 software using the maximum likelihood (ML) method. Bootstrap analysis was performed with 1000 replicates. The virus A/Guangxi/01591/2024(H10N3) reported in Guangxi is marked with black triangles. The other 2 viruses isolated from human in People's Republic of China were marked with purple circle. Scale bar indicates nucleotide substitutions per site.
